# Local tree cover predicts mosquito species richness and disease vector presence in a tropical countryside landscape

**DOI:** 10.1007/s10980-025-02105-0

**Published:** 2025-05-28

**Authors:** Johannah E. Farner, Meghan Howard, Jeffrey R. Smith, Christopher B. Anderson, Erin A. Mordecai

**Affiliations:** 1 Department of Biology, Stanford University, 317 Jane Stanford Way, Stanford, CA USA; 2 Department of Ecology and Evolution, Princeton University, 106A Guyot Ln, Princeton, NJ USA; 3 Planet Labs, 645 Harrison St, San Francisco, CA USA

**Keywords:** Mosquitoes, Culicidae, Community assembly, Tree cover, *Aedes albopictus*, Biodiversity

## Abstract

**Context:**

Land use change and deforestation drive both biodiversity loss and zoonotic disease transmission in tropical countrysides. For mosquito communities that can include disease vectors, forest loss has been linked to reduced biodiversity and increased vector presence. The spatial scales at which land use and tree cover shape mosquito communities present a knowledge gap relevant to both biodiversity and public health.

**Objectives:**

We investigated the responses of mosquito species richness and *Aedes albopictus* disease vector presence to land use and to tree cover surrounding survey sites at different spatial scales. We also investigated species compositional turnover across land uses and along environmental gradients.

**Methods:**

We paired a field survey of mosquito communities in agricultural, residential, and forested lands in rural southern Costa Rica with remotely sensed tree cover data. We compared mosquito richness and vector presence responses to tree cover measured across scales from 30 to 1000 m, and across land uses. We analyzed mosquito community compositional turnover between land uses and along environmental gradients of tree cover, temperature, elevation, and geographic distance.

**Results:**

Tree cover was both positively correlated with mosquito species richness and negatively correlated with the presence of the common invasive dengue vector *Ae. albopictus* at small spatial scales of 90–250 m. Land use predicted community composition and *Ae. albopictus* presence.

**Conclusions:**

The results suggest that local tree cover preservation and expansion can support mosquito species richness and reduce disease vector presence. The identified spatial range at which tree cover shapes mosquito communities can inform the development of land management practices to protect both ecosystem and public health.

**Supplementary Information:**

The online version contains supplementary material available at 10.1007/s10980-025-02105-0.

## Introduction

Humans have modified more than half of Earth’s land surface through activities such as deforestation, agricultural intensification and expansion, and urbanization (Hooke et al. 2012). Land use and land cover change fundamentally alter ecosystems, with strong impacts on biodiversity including species endangerment, loss, and invasion (e.g., Sala 2000; Pereira et al. 2010; Maxwell et al. 2016; Giam 2017). For communities containing species that transmit human diseases, the impacts of land use change on biodiversity can also impact human health. For example, the growing land area used for pineapple production in Costa Rica corresponds to an increase in highly suitable habitat for the malaria vector *Anopheles albimanus* (Rhodes et al. 2022). Developing land management solutions that benefit both biodiversity and public health is particularly important for countryside landscapes characterized by natural habitat remnants patchworked with human residential and agricultural infrastructure. These landscapes are globally dominant, are disproportionately impacted by land use change and zoonoses, and are critical to biodiversity conservation (Norris 2008; Chazdon et al. 2009; Maxwell et al. 2016; Sokolow et al. 2022). Percent tree cover at small spatial scales of < 100 m has emerged as a reliable predictor of biodiversity for taxa in Latin American tropical countrysides including birds, non-flying mammals, and bats (Mendenhall et al. 2016), suggesting that local tree cover management holds promise as a practicable conservation tool. However, major knowledge gaps remain surrounding how local tree cover relates to invertebrate biodiversity, including for arthropod vectors of human diseases.

Mosquito communities (family Culicidae) are relevant to biodiversity and public health in countryside landscapes because they are sensitive to tree cover and habitat type, act as prey, predators, and detritivores in aquatic and terrestrial food webs (Addicott 1974; Heard 1994; Daugherty et al. 2000; Poulin et al. 2010), and can include species that are important vectors of diseases including malaria, dengue, chikungunya, Zika, yellow fever, West Nile fever, and arboviral encephalitis (Garmendia et al. 2001; Lemon et al. 2008; LaPointe et al. 2012; World Health Organization 2021). Previous studies show associations between forest conversion and low mosquito biodiversity, and suggest that high rates of land use change and active invasions by major vector species are together reshaping mosquito communities in ways that increase disease risk (Ferraguti et al. 2016; Meyer Steiger et al. 2016; Burkett-Cadena and Vittor 2018; Chaves et al. 2021b). These patterns are likely shaped by abiotic and biotic conditions associated with tree cover and land use context that affect the presence and abundance of mosquito species that vary in their thermal niches, aquatic breeding habitat requirements, and preferred groups of vertebrates for blood meals (Laird 1988; Gutman et al., 2004; Mordecai et al. 2019; Prevedello et al. 2019). Clarifying the spatial scales at which these landscape features shape mosquito communities is critical to understanding how tree cover management might balance biodiversity conservation, public health, and economic needs.

The countryside of Costa Rica is an ideal system in which to study relationships between land cover, mosquito community characteristics, and disease vector occurrence. This region, like neighboring Latin American countries, has a large burden of mosquito-borne diseases, including dengue virus, with two invasive *Aedes* spp. vectors potentially contributing to transmission (Rezza 2012; Kraemer et al. 2015; World Health Organization 2021). *Aedes aegypti* is considered the primary dengue vector in Costa Rica, but the ongoing, patchily described invasion by the globally important vector species *Aedes albopictus* is potentially reshaping disease risk (Troyo et al. 2006; Calderón-Arguedas et al. 2010, 2015; Rojas-Araya et al. 2017). Globally, *Ae. albopictus* and *Ae. aegypti* are predominantly associated with rural and urban human settlements, respectively, suggesting that *Ae. albopictus* and its responses to tree cover may play particularly important roles in countryside dengue transmission (Braks et al. 2003; Tsuda et al. 2006). Additionally, intensive long-term research in this system has characterized many links between landscape context, biodiversity, and ecosystem services (e.g., Daily et al. 2001; Ricketts et al. 2004; Karp et al. 2013; Frank et al. 2017; Hendershot et al. 2020; Langhans et al. 2022), but the corresponding links to mosquito biodiversity and disease vector presence remain undescribed.

Here, we combine field observations of mosquito communities in forested, agricultural, and residential settings in a rural area of southern Costa Rica with remotely-sensed land cover data in order to investigate mosquito community responses to forest cover and land use. We ask: How do mosquito community characteristics vary with tree cover measured at different spatial scales, among land use types, and along environmental gradients? We hypothesize that lower local tree cover and non-forest land uses are associated with lower mosquito species richness but higher presence of human-associated *Aedes* spp. vectors. 

## Methods

### Study area

**Fig. 1:**
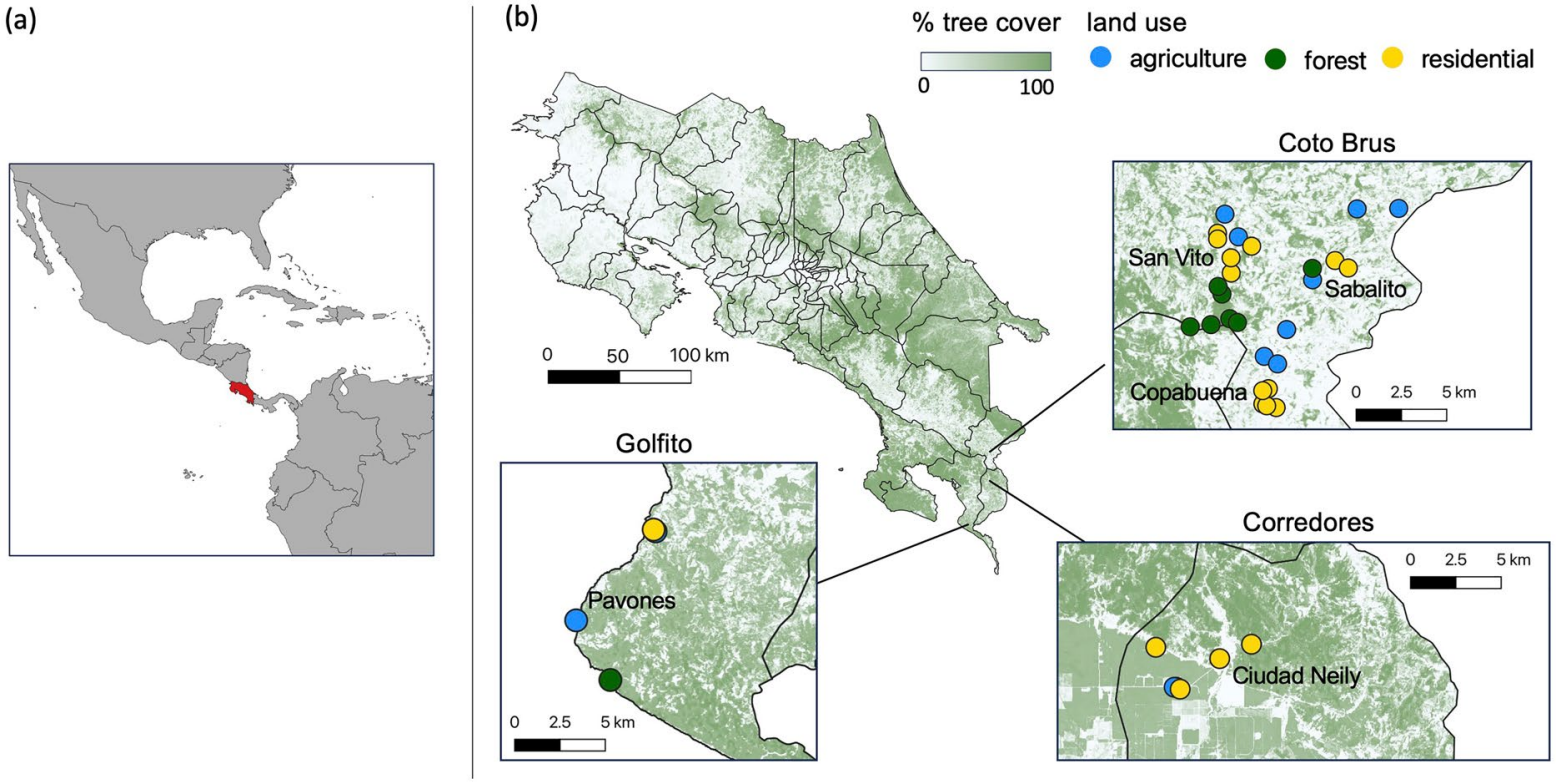
Tree cover and land use varied across study sites in Costa Rica. **a** Location of Costa Rica in Central America is highlighted in red. **b** Map of Costa Rica with canton boundaries delineated by lines, tree cover (remotely sensed at 30 m resolution) indicated in shades of green, and sampling sites indicated by colored points within the three insets. Insets shows study sites within Golfito, Corredores, and Coto Brus cantons. Blue, green, and yellow points denote agricultural, forest, and residential land uses, respectively. Text labels within insets denote districts

This study was conducted in the cantons of Coto Brus, Corredores, and Golfito (8°43′14″N, 82°57′20″W), located in the southern Puntarenas region of Costa Rica along the border with Panama (Fig. 1a). The region ranges from coastal lowland tropical rainforest (0 m above sea level) to high elevation cloud forest (1500 m above sea level) and has distinct wet and dry seasons. The study area is predominately composed of rural communities surrounded by agriculture interspersed with forest patches, and also includes the protected Las Cruces forest reserve. In this area, dengue is endemic, *Ae. aegypti* is common, and *Ae. albopictus* has a growing presence (World Health Organization 1994; Troyo et al. 2008, 2009; Rojas-Araya et al. 2017).

### Study sites

With landowner permission, we accessed 37 sites representing three broad land use classes that were determined on-site by the survey team: residential (N = 17), agricultural (N = 12), and forest (N = 8) (Fig. 1b). In the Coto Brus canton (N = 12 residential sites, 8 agricultural, 7 forest), sites were in the districts San Vito, Sabalito, and Copabuena; in Corredores canton (N = 4 residential, 2 agricultural), sites were in Ciudad Neily district; in Golfito canton, (N = 1 residential, 2 agricultural, 1 forest), sites were in Pavones district. Residential site collections took place in the yards of residences in urban and peri-urban areas; agricultural site collections took place in the agricultural fields of coffee plantations, an oil palm plantation, a pine plantation, one mixed crop field, and one pasture; forest site trapping took place in primary and secondary forest edges, interiors, and fragments, on both protected and unprotected lands.

### Environmental variables

To quantify percent tree cover, we used a 30 m resolution map of tree cover in Costa Rica created by Echeverri et al. (2022) from multi-sensor satellite observations (2005–2017) and fine-scale tree cover maps (Fig. 1a). From this map, we calculated percent tree cover at different spatial scales surrounding each site using the R package “raster” (Hijmans et al. 2015). Specifically, we began by calculating percent tree cover within a radius of 30 m; we then increased this radius by increments of 10 m up to 200 m, and by increments of 50 m for radii between 200 and 1000 m (following Mendenhall et al. 2014). To account for mosquito interspecific variation in sensitivity to thermal conditions (Mordecai et al. 2019), which could affect observed relationships between land cover and mosquito community characteristics along the 1500 m elevational gradient surveyed, we additionally extracted mean annual temperature data from 1970 to 2000 for each study site from the WorldClim 1 km^2^ resolution mean annual temperature dataset (Fick and Hijmans 2017).

### Sample collection

We trapped mosquitoes during the rainy season, visiting all sites twice between June 19 and August 9, 2017, excepting the four sites in Pavones, which were trapped once (N = 70 trap nights). The interval between collections at a given site ranged from 8 to 36 days (mean = 22 days, SD = 8 days) (Table S1). In order to detect mosquito species with a variety of habitat and blood meal host preferences, we used a mix of trapping and manual collection methods (Thongsripong et al. 2013; Hoshi et al. 2014; Giordano et al. 2020; Romero-Vega et al. 2023). Specifically, at each site, we placed a total of four traps overnight for a 12–16 h period within an area of 30 m in radius: one unlighted CDC trap baited with carbon dioxide produced by a mixture of Fleischmann’s Dry Active Yeast, household refined sugar, and water to mimic vertebrate respiration; one BG Sentinel baited with a BG-Lure and octanol to attract human-specialist mosquitoes; and two BG-GAT traps furnished with yellow sticky cards, corn oil, and a mixture of water and local leaf litter to attract gravid female mosquitoes. Trap locations within sites were chosen per BioGents recommendations, and square metal frames covered in large black plastic bags were placed over BG Sentinel and BG-GAT traps for protection from rainfall. To supplement the overnight trapping, during each trapping session, mosquito larvae were collected from breeding habitats and one of three trained members of the field team carried out 20 min of direct aspiration, over a standardized time and area.

### Mosquito identification

Four field team members trained to morphologically identify *Ae. albopictus* and *Ae. aegypti* picked out, sexed, and counted individuals of these species collected at each site. All morphological identifications were confirmed by the field team lead to ensure consistency. All other mosquitoes were counted, stored, and transported to Stanford University for molecular identification. We extracted and amplified DNA from the mitochondrial CO1 gene from pooled samples of mosquitoes from each trap night at each site (N = 70 pools) using MyTaq RedMix (Meridian Bioscience, Cincinnati, OH), following the protocol provided by the manufacturer. Amplified DNA libraries were prepared for next-generation sequencing with Nextera (Illumina, Inc., San Diego, CA) and sequenced via Illumina MISEQ, with samples containing *Aedes albopictus* and *Culex tarsalis* DNA as positive controls. We removed primer sequences and paired forward and reverse reads from the sequencing data with the R package “dplyr”, and then used the R package “dada2” to filter and trim the DNA sequences to 473 bp, with a minimum overlap of 20 bases and a maximum of five expected errors (Callahan et al. 2016; Wickham et al. 2023). We estimated taxonomic placement for the sequenced mosquitoes by using the R packages “Biostrings” and “DECIPHER” to group DNA sequences into operational taxonomic units (OTUs, henceforth referred to as species) of 97% sequence similarity, and comparing representative sequences for each species to the BOLD and GenBank database records (Altschul et al. 1990; Ratnasingham and Hebert 2007; Wright 2016; Pagès et al. 2022). Species were identified based on top matches with sequence similarity ≥ 97% (Hardulak et al. 2020). When sequence similarity to the top match was < 97%, a higher level of taxonomic identification (e.g., genus) was assigned based on placement within a phylogenetic tree of the BOLD database sequences.

### Statistical analyses

We described mosquito communities in terms of species richness and species composition by combining presence data from the morphologically identified *Aedes* data and the sequencing data. To assess the completeness of species pool sampling for each land use type, we plotted species accumulation curves with the function “accumcomp” in the package BiodiversityR (Kindt and Coe 2005). To quantify relationships between species richness and percent tree cover, we used generalized linear models (GLMs) with negative binomial error corrections for overdispersion and mean-centered independent variables. To assess the spatial scales at which tree cover best predicted species richness, we compared AIC values for GLMs that included percent tree cover surrounding each site calculated at radii ranging from 30 to 1000 m. We used binomial logistic regression to analyze relationships between *Ae. albopictus* disease vector presence/absence and percent tree cover across spatial scales. For the 1000 m spatial scale where climate data were available, we additionally assessed the relative influence of mean annual temperature on species richness and *Aedes* vector presence with GLMs including mean annual temperature and its interaction with tree cover.

To compare species richness and *Aedes* vector presence between forest, agricultural, and residential land uses, we used Kruskal–Wallis tests with Bonferroni p-value adjustments to account for multiple comparisons.

To compare species composition among land use types and along environmental gradients, we first calculated the Jaccard coefficient of community similarity for each pair of sites for use in statistical tests and ordination. We then tested for differences in community similarity among land uses with permutational analysis of variation (PERMANOVA), first for all land use types, and then with pairwise adonis functions. Because PERMANOVA is sensitive to heterogeneity in dispersion among groups (Anderson and Walsh 2013), we additionally tested whether dispersion differed among land use types using Tukey’s Honest Significant Differences method with betadisper() calculations of group average distances to the median. To visualize community similarity across land uses, we used non-metric multidimensional scaling (NMDS). All the above analyses of compositional similarity among land uses were run using the R package “vegan” (Oksanen et al. 2013); for the pairwise PERMANOVA analyses, we used the R package “ecole” which provides wrapper functions for “vegan” (Smith 2022). Finally, to quantify compositional turnover along environmental gradients of tree cover, mean temperature, elevation, and geographic distance, we used the R package “gdm” for generalized dissimilarity modeling (GDM), a form of nonlinear matrix regression that is robust to collinearity (Fitzpatrick et al. 2022). As above, we compared GDM models that incorporated tree cover at radii ranging from 30 to 1000 m to identify the spatial scale at which tree cover best explained compositional turnover.

All analyses were performed in R version 4.2.1. In addition to the R packages cited above, we used the packages “tidyverse” (Wickham et al. 2019), “cowplot” (Wilke et al. 2019), “MASS” (Ripley et al. 2013), “interactions” (Long and Long 2019), “gridExtra” (Auguie et al. 2017), and “reshape2” (Wickham 2007) for data analysis and figure generation.

## Results

### Environmental variables

Across sites, tree cover ranged from 0 to 100% (mean = 27.9%, SD = 34.8%) at the smallest spatial scale we considered, a 30 m radius. Surrounding tree cover within a 1000 m radius, the largest spatial scale considered, ranged from 8.2% to 74.8% (mean = 33.4%, SD = 20.3%). The lowest and highest site elevations were 12 m and 1451 m above sea level, respectively (mean = 776 m, SD = 473.2 m). Mean annual temperature ranged from 18.8 to 26.4 ℃ (mean = 22.6 ℃, SD = 2.3 ℃).

### Mosquito collections

**Fig. 2:**
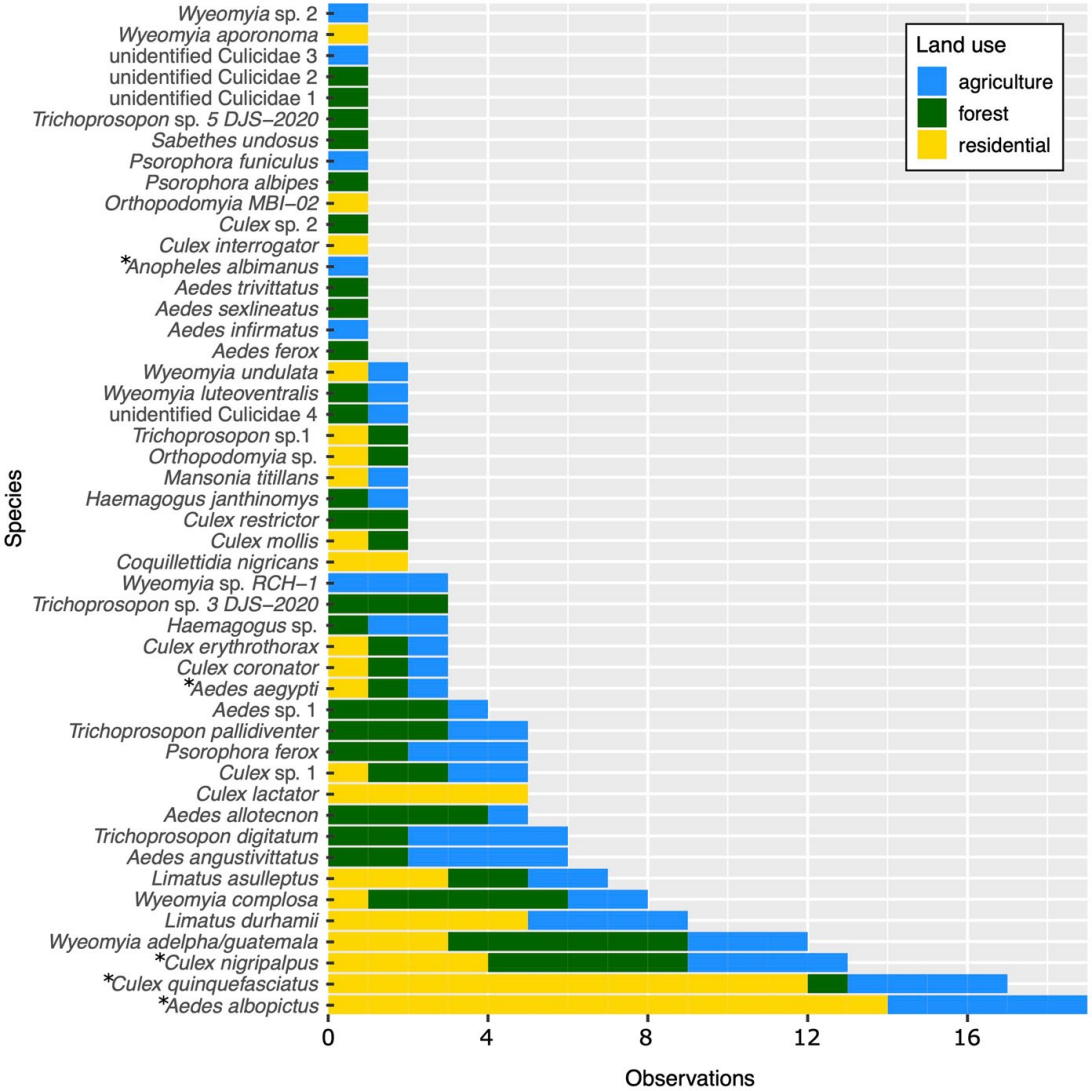
The commonness of 48 observed mosquito species varied both overall and among land use types. Blue bars show the number of agricultural sites in which each species was present, green bars show forest sites, and yellow bars show residential sites. Asterisks indicate species known to vector human diseases

A total of 1,283 mosquitoes representing 48 species in 13 genera were collected from 37 sites (Fig. 2, Table S2). The number of mosquitoes collected at a site ranged from one to 244 (mean = 35, SD = 64) (Table S1). Of these, 99 individuals from 14 residential and five agricultural sites were morphologically identified as *Ae. albopictus*, and five total *Ae. aegypti* individuals were identified from one forest, one agricultural, and one residential site. *Ae. albopictus* DNA was detected in the pooled samples of molecularly identified mosquitoes from seven of the 19 sites where *Ae. albopictus* individuals were also morphologically identified, and no sites without morphologically identified specimens. The five most common species were *Ae. albopictus*, *Culex quinquefasciatus*, *Cx. nigripalpus*, *Wyeomyia adelpha/Guatemala*, and *Limatus durhamii* (Fig. 2, Table S3).

#### Species richness

Site-level species richness ranged from one to 19 (mean = 4.89, SD = 4.1) (Table S2). Overall species counts for forest, agricultural, and residential land uses were 33, 29, and 21, respectively. Ten species (21%) were observed in all three land uses. Nineteen species (40%) were shared among forest and agricultural land uses, 13 species (27%) were shared among agricultural and residential land uses, and 12 species (25%) were shared among forest and residential land uses (Fig. 2, Table S3). Eleven species (23%) were found only in forested settings, six species (13%) were found only in agricultural settings, and six species (13%) were found only in residential settings (Fig. 2, Table S3). Two species, *Ae. albopictus* and *Culex quinquefasciatus*, were common (observed at > 50% of sites) in residential settings, no species were common in agricultural settings, and three species—*Culex nigripalpus*, *Wyeomyia complosa*, and *Wyeomyia adelpha/guatemala*—were common in forested settings (Table S1). Species accumulation curves indicate that more species would have been observed in each land use class with additional sampling, but at a lower rate in residential compared to forest and agricultural land uses (Figure S1).

#### Disease vectors

At least five of the mosquito species observed are known vectors of human diseases. Three of these—the dengue and chikungunya virus vector *Ae. albopictus* (present at 19 sites) and the St. Louis Encephalitis virus vectors *Cx. quinquefasciatus* (present at 17 sites) and *Cx. nigripalpus* (present at 13 sites)—were the three most frequently observed species (Reisen 2003; Simmons et al. 2012). Rarely observed vector species included the dengue, chikungunya, yellow fever, and Zika virus vector *Ae. aegypti* (present at three sites spanning all three land use types) and the malaria vector *Anopheles albimanus* (present at one agricultural site) (Zimmerman 1992; Simmons et al. 2012). In contrast to *Ae. aegypti*, *Cx. nigripalpus,* and *Cx. quinquefasciatus*, which were observed in all land use types, *Ae. albopictus* was observed only in residential and agricultural settings associated with intensive human modification.

#### Model results

**Fig. 3:**
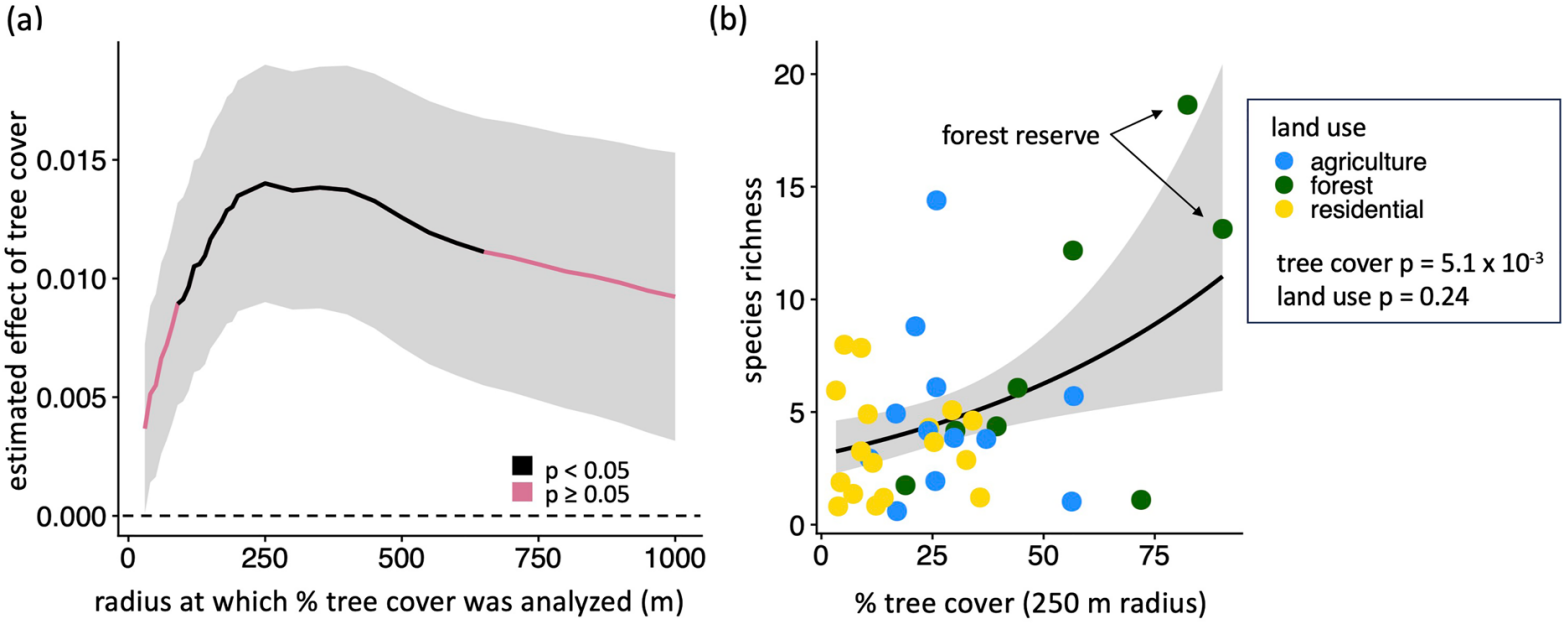
Species richness is correlated with tree cover surrounding survey sites for radii between 90 and 650 m. **a** The estimated effect of surrounding tree cover calculated across spatial scales on species richness. Radii where the relationship between tree cover and species richness is significant (p < 0.05) are shown in black; others are shown in pink. Values above the dashed line are positive. **b** Species richness increases with percent tree cover at a 250 m radius: the scale identified as having the strongest effect. Land use (colored points) and species richness are not significantly correlated. The site with the highest species richness and the site with the highest surrounding tree cover were both located in the Las Cruces forest reserve (arrows). In both panels, gray shading shows ± 1 SE

Mosquito species richness was explained by tree cover, but not by land use type. Comparisons of GLMs using tree cover calculated for radii ranging between 30 and 1000 m surrounding each site indicated that species richness was positively correlated with tree cover at radii between 90 and 650 m, and tree cover at a 250 m radius had the largest effect size (estimated effect = 1.40 × 10^−2^, SE = 5.00 × 10^−3^, z-value = 2.8, p-value = 5.08 × 10^−3^) (Fig. 3a, Table S4). At the 1000 m spatial scale where both tree cover and climate data were available, the interaction between tree cover and mean annual temperature had a significant effect on species richness (estimated effect = − 9.48 × 10^−3^, SE = 3.48 × 10^−3^, z-value = − 2.72, p-value = 6.48 × 10^−3^ (Table S5). Specifically, at high temperatures, species richness was low even when tree cover was high (Figure S2). By contrast, Kruskal–Wallis test results indicated that species richness did not differ significantly among forested, agricultural, and residential sites (chi-squared = 2.84, df = 2, p-value = 0.24) (Fig. 3b). Notably, the highest species richness was observed at a site in the Coto Brus forest reserve (Fig. 3b).

#### Relationships of *Ae. albopictus* presence to tree cover and land use

From the *Aedes* disease vector survey, we present results only for *Ae. albopictus* because observations of *Ae. aegypti* were insufficient for statistical analysis. Both tree cover and land use type predicted *Ae. albopictus* presence. Comparisons of GLMs using tree cover calculated for radii ranging between 30 and 1000 m surrounding each site indicated that *Ae. albopictus* presence was negatively correlated with tree cover at radii between 30 and 250 m, and was best explained by tree cover at a 110 m radius (estimated effect = − 4.46 × 10^−2^, SE = 1.83 × 10^−2^, z-value = − 2.44, p-value = 1.47 × 10^−2^) (Fig. 4a, Table S6). At the 1000 m spatial scale where we additionally assessed the influence of climate, *Ae. albopictus* presence was negatively correlated with tree cover and positively correlated with temperature (tree cover estimated effect = − 8.34 × 10^−2^, SE = 3.61 × 10^−2^, z-value = − 2.31, p-value = 2.08 × 10^−2^; mean annual temperature estimated effect = 9.36 × 10^−1^, SE = 4.2 × 10^−1^, z-value = 2.23, p-value = 2.5 × 10^−2^) (Figure S3, Table S7). Land use type also predicted *Ae. albopictus* presence (Kruskal–Wallis chi-squared = 15.02, p-value = 5.48 × 10^−4^). Specifically, *Ae. albopictus* was significantly more likely to be observed in residential settings (present at 14/17 sites) than in forested settings (present at 0/8 sites) (Kruskal–Wallace chi-squared = 14.37, Bonferroni-adjusted p-value = 4.49 × 10^−4^), and its presence in agricultural settings (present at 5/12 sites) did not differ significantly compared to either residential (Kruskal–Wallace chi-squared = 4.98, adjusted p-value = 7.8 × 10^−2^) or forested (Kruskal–Wallace chi-squared = 4.22, adjusted p-value = 1.20 × 10^−1^) settings. Eighteen of the 19 sites where *Ae. albopictus* was present were surrounded by < 35% tree cover within a 110 m radius. The only site within a pine plantation was a clear outlier, where *Ae. albopictus* was present under 75% tree cover (Fig. 4b).

**Figure:**
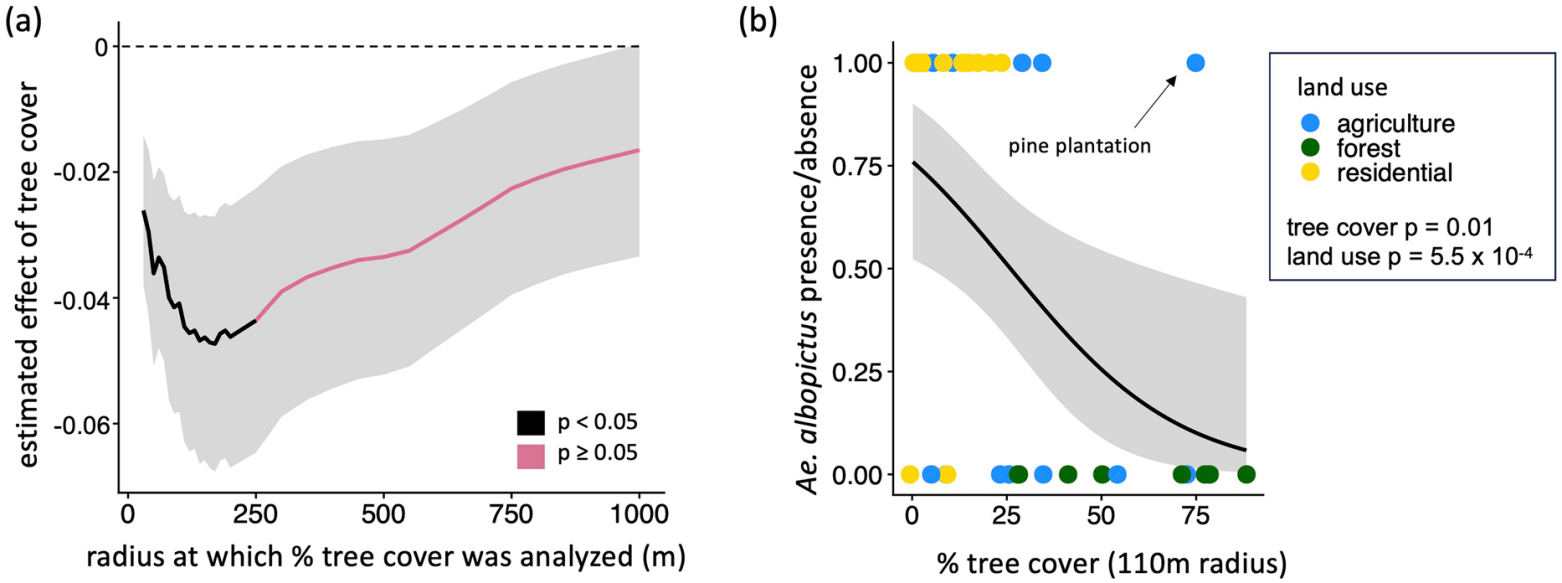
*Aedes albopictus* is most likely to be found at low tree cover levels and in residential settings. **a** The estimated effects of tree cover across spatial scales on *Ae. albopictus* presence, where black lines indicate statistically significant (p < 0.05) effects and pink lines indicate non-significant (p > 0.05) relationships. Values below the dashed line are negative. **b** Land use type (colored points), percent tree cover, and *Ae. albopictus* presence/absence at the highest-significance 110 m radius. In both panels, gray shading shows ± 1 SE

#### Species composition across land uses

**Fig. 5:**
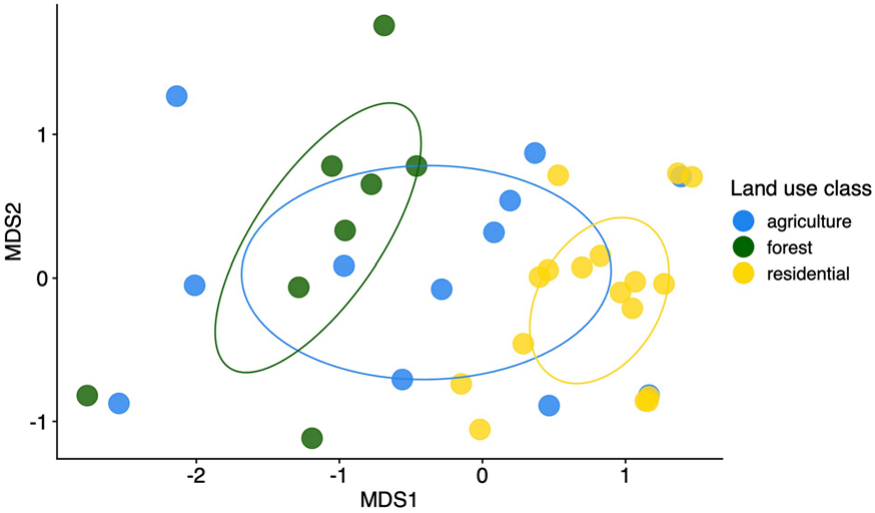
Distinct mosquito communities were observed in forest and residential land uses, while communities in agricultural settings overlapped with both other land use types. NMDS ordination visualization groups sites by community similarity, with colors indicating land use types (blue: agriculture, green: forest, yellow: residential). Each point represents the community at one study site, and the distance between points is smaller for more similar communities. Ellipses show 95% confidence intervals for ordination of agriculture, forest, and residential mosquito communities

In contrast to species richness, community composition and dispersion were predicted by land use type. PERMANOVA results comparing all three land uses showed that land use significantly affected community composition (sum of squares = 2.24, R^2^ = 0.16, F-value = 3.24, p-value = 0.001), and pairwise PERMANOVAs showed that agricultural and forest mosquito communities differed significantly from residential communities (Table 1). Wider dispersion among agricultural compared to residential mosquito communities (average distance to the median: agriculture = 0.618, forest = 0.572, residential = 0.496; Tukey test adjusted p-values: residential—agricultural = 0.0104, residential—forested = 0.218, forested—agricultural = 0.604) likely contributed to the community dissimilarity detected between these land uses (Anderson and Walsh 2013). Supporting these statistical results, NMDS visualization of communities grouped by land use type highlights that agricultural communities bridge distinct forest and residential communities (NMDS stress = 0.12) (Fig. 5). The wider variation among agricultural sites is also evident from the species observation table: no single species was observed at more than 1/3 of all agricultural sites, whereas *Ae. albopictus* and Unidentified Culicidae 1 were both observed at > 70% of residential sites, and *Culex nigripalpus*, *Wyeomyia adelpha/guatemala*, and *Wyeomyia complosa* were each observed at > 60% of forested sites (Tables S2, S3). On the NMDS plot, forest land use sites falling far outside of the 95% CI included the single forest fragment and two of four forest edges surveyed (Figure S4). Agricultural land use outliers clustering near the residential group included both oil palm plantations surveyed and one coffee plantation, and those clustering nearer the forest group included three coffee plantations (Figure S4). Each site in the geographically distinct Pavones district that was sampled only once fell outside of the 95% CIs for its land use type (Figure S4).

**Table 1:** PERMANOVA results for community composition compared among land use types

Land use pair	Sum of squares	F-Value	R^2^	Bonferroni-adjusted p value
Agriculture vs. forest	0.645	1.59	0.0813	0.117
Agriculture vs. residential	0.846	2.52	0.0853	0.018*
Forest vs. residential	1.82	5.87	0.203	0.003*

*p < 0.05

#### Species turnover along environmental gradients

**Fig. 6:**
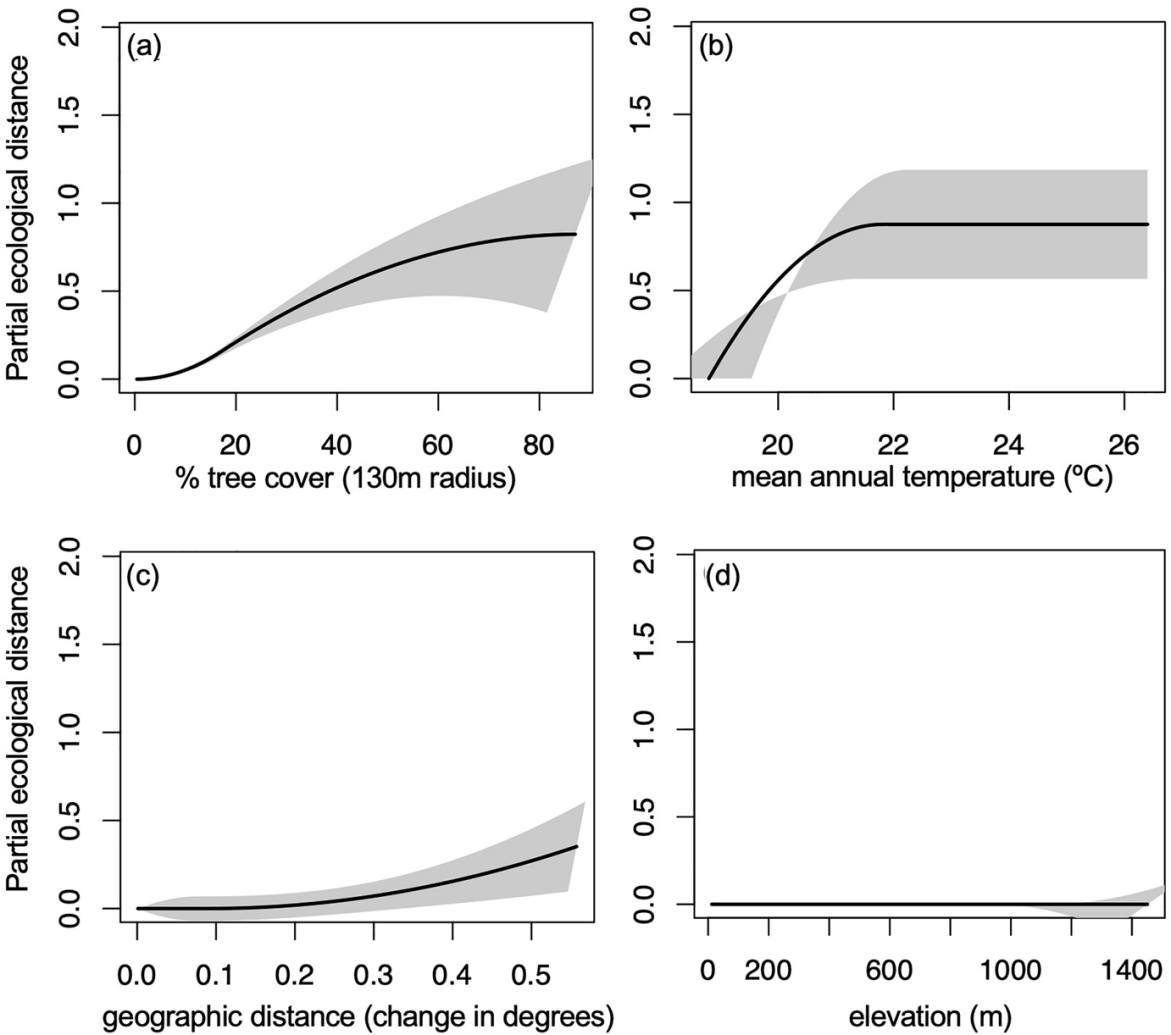
Generalized dissimilarity modelling (GDM) of community dissimilarity indicated that **a** percent tree cover and **b** mean annual temperature explained 7% of deviation from the null. **c** Geographic distance contributed minimally, and **d** elevation did not significantly contribute to community turnover. In each panel, the x-axis shows the environmental gradient and the y-axis shows the amount of compositional turnover, measured as partial ecological distance. The maximum height the spline reaches on the y-axis indicates the total amount of compositional turnover the gradient is associated with, and the slope shows how the rate of compositional turnover varies along the environmental gradient. The difference in height between any two points along the I-spline corresponds to the modeled contribution of that predictor variable to the difference between those points. Grey shading shows ± 1 standard deviation when 70% of sites are sampled ten times

Finally, generalized dissimilarity modeling (GDM) indicated that environmental gradients explained little of the species turnover among sites. Among the spatial scales for which tree cover was calculated, the model using the 130 m radius explained the highest amount of species turnover among sites (Table S8). The model that included mean annual temperature, geographic distance, and tree cover at the 130 m radius explained 7% of species turnover among sites. Elevation showed no relationship with species turnover. Whereas increasing tree cover was associated with a consistent increase in community turnover, increasing temperature was associated with a steep increase in community turnover up to a plateau around 22 ℃, and increasing geographic distance was associated with comparatively limited turnover (Fig. 6).

## Discussion

We found that local tree cover, but not land use (residential, agricultural, or forest), predicted mosquito species richness, suggesting that more diverse communities occur at higher tree cover. By contrast, community composition was more predictable for forested and residential land uses, and more variable among agricultural sites, but environmental gradients of tree cover, climate, and geographic distance explained only 7% of species turnover among sites. *Ae. albopictus* presence varied significantly with both tree cover and land use, but in the opposite direction from mosquito richness: increasing with lower tree cover and in residential compared to forested sites (with intermediate probability in agricultural sites). Overall, our results add to support from both mosquitoes and other taxa that natural and semi-natural habitat, including tree cover, sustains substantial biodiversity and ecosystem services—here, in the form of protection against mosquitoes that vector human disease (Millennium Ecosystem Assessment 2005; Jose 2009; Mendenhall et al. 2014, 2016; Frank et al. 2017; Barrios et al. 2018; Burkett-Cadena and Vittor 2018; Frishkoff et al. 2019; Langhans et al. 2022; Perrin et al. 2022).

### Mosquito richness and species composition

The spatial scales at which tree cover predicted mosquito species richness were small (90–650 m), and comparable to previous findings for other taxa in the same study area, highlighting the disproportionately positive impact of small patches of trees on biodiversity (Mendenhall et al. 2014, 2016; Frank et al. 2017). The radius at which tree cover best predicted species richness was 250 m; by comparison, biodiversity was correlated with tree cover at small spatial scales for non-flying mammals (70 m), bats (50–60 m), birds (30 m), reptiles (50 m), and amphibians (80 m) in the same region of Costa Rica (Mendenhall et al. 2014, 2016; Frank et al. 2017). Although the radius at which mosquito species richness responded most strongly to tree cover was larger compared to previously studied taxa, the spatial scale remained local, and significant effects of tree cover were found at radii as small as 90 m. Our observation of a positive relationship between species richness and tree cover aligns with those of many other studies of mosquito diversity along land cover gradients in locations including Latin America, Asia, and Europe (e.g., Johnson et al. 2008; Thongsripong et al. 2013; Ferraguti et al. 2016; Chaverri et al. 2018), and our analysis of the distance around sampling sites at which tree cover shapes mosquito communities contributes to clarifying the spatial scales at which mosquitoes respond to landscape features.

Our observation that mosquito community composition was distinct among different land uses is consistent with patterns observed both for other taxa in this system, and for mosquitoes in other regions (Mendenhall et al. 2014, 2016; Meyer Steiger et al. 2016; da Silva Pessoa Vieira et al. 2022). In agricultural settings, relatively high species richness and community similarity with forested settings support the argument that farmlands can contribute substantially to biodiversity (e.g., Norris 2008). However, the high proportion of species unique to forest habitats and the high species richness observed inside the large Las Cruces forest reserve also reaffirm the importance of forests and protected areas as refugia for biodiversity (Coetzee et al. 2014; Mendenhall et al. 2016). Additionally, the compositional variability among agricultural settings and the close community resemblance between some agricultural and residential sites indicate a need for additional research on how mosquito communities respond to specific land uses, crop assemblages, or management practices that can result in similar levels of tree cover. For example, organic farming methods are associated with higher arthropod diversity globally compared to conventional methods, and Kenyan ricelands that rely on natural rather than artificial irrigation have higher mosquito species richness (Muturi et al. 2006; Lichtenberg et al. 2017). Costa Rican croplands that are less intensively farmed support greater bird species richness, a pattern that may also hold for mosquitoes (Hendershot et al. 2020).

Environmental gradients of tree cover and temperature shaped species turnover, but explained only 7% of variance in community composition, suggesting that additional habitat characteristics may play important roles in determining species composition. Such factors might include local microclimates, differences among types of tree cover (e.g., agricultural types, primary versus secondary forest), and/or the presence of vertebrate hosts preferred by different mosquito species. Differences in species abundances and community evenness, which were not quantified here, might also respond more strongly to gradual environmental change than the identities of the species present. Additionally, undersampling of communities may have contributed to this result by limiting the repeatability of community composition observed at environmentally similar sites. However, our result that land use predicts species composition, while land cover predicts species richness, aligns with patterns of abundance-based Dipteran and Culicid diversity observed in the tropical Australian countryside (Smith and Mayfield 2015; Meyer Steiger et al. 2016).

### Disease vectors

The most frequently observed disease vector, *Ae. albopictus*, was more likely to be observed in sites with lower surrounding tree cover and agricultural or residential land uses, suggesting that rural landscapes with more forest and tree cover may be more resistant to invasion by this species. These observations align with this species’ well-established preferences for taking blood meals from humans and livestock (Niebylski et al. 1994; Richards et al. 2006), and its association with rural, agricultural, suburban, and/or deforested settings in the Americas, Asia, and Africa (Gilotra et al. 1967; Braks et al. 2003; Young et al. 2017; Câmara et al. 2020; Canelas et al. 2023). The 30–250 m radii at which tree cover negatively affected *Ae. albopictus* presence fell within the 90–650 m range at which tree cover positively affected species richness, suggesting promise for local tree cover management as a means of supporting both public health and biodiversity conservation. Protection against mosquito disease vectors conferred by tree cover may extend beyond *Ae. albopictus* to include at least 16 other significant vectors of human diseases that are favored by deforestation, including *Ae. aegypti,* multiple Anopheline malaria vectors, and the Amazonian malaria vector *Nyssorhynchus darlingi* (Burkett-Cadena and Vittor 2018; Chaves et al. 2021a, b).

Our finding that *Ae. albopictus* was associated with, but inconsistently observed in, agricultural settings (present in 33% of agriculture sites) reinforces that agricultural lands have the potential to either harbor or resist invasive species, and suggests vector associations with agricultural subtypes as a key future research direction. In our survey, factors that differentiated the high tree-cover pine plantation and two of the six surveyed coffee plantations as suitable habitat for *Ae. albopictus* are of particular interest. For example, *Ae. albopictus* larvae were found in banana leaves and stumps in a shade-grown coffee plantation. Understanding vector responses to agriculture is particularly important because this land use is the most likely candidate for local tree cover management in Costa Rica due to its spatial extent, the established Payment for Ecosystem Services program for incentivizing landowner forest retention and tree planting, and a previous finding that urban tree cover is correlated with dengue incidence in this region, while forest cover at the district level is negatively associated with dengue hospitalizations and outbreaks (Sánchez-Azofeifa et al. 2007; Troyo et al. 2009; Piaggio et al. 2024).

The findings of this study are subject to several limitations resulting from less extensive sampling of the mosquito community. Extensive sampling is required to fully capture the high arthropod biodiversity present in tropical areas (Coddington et al. 2009), such that unsaturated species accumulation curves are common in these systems (e.g., Novotný and Basset 2000; Responte and Nuneza 2016; Thormann et al. 2016; Romero-Vega et al. 2023; Kirmse 2024). Many more mosquito species may have been observed with increased collection time at each site, particularly with additional coverage to include the dry season, as highlighted by seasonal mosquito species richness and abundances observed in Costa Rica by Romero-Vega et al. (2023). The sequencing approach we took to identify species other than *Ae. albopictus* and *Ae. aegypti* allowed us to efficiently identify specimens compared to a traditional morphological approach, but was limited by sequencing success and the availability of database entries for comparison. Additionally, the pooling of sequenced mosquitoes by site and trap night prevented measurements of species abundances and evenness, which are likely sensitive to tree cover and are key indicators of community structure and functioning, including the potential for vector species to transmit diseases (Franklinos et al. 2019). Despite these limitations, the number of mosquitoes collected and species richness we observed are on par with other mosquito studies in Costa Rica (Calderón-Arguedas et al. 2008; Burkett-Cadena et al. 2013; Chaverri et al. 2018; Romero-Vega et al. 2023), and the statistically significant environmental responses of mosquito community characteristics were consistent with hypotheses grounded in the findings of other studies with much larger sample sizes (e.g., Braks et al. 2003; Johnson et al. 2008; Chaves et al. 2021a, b; da Silva Pessoa Vieira et al. 2022). We hypothesize that additional sampling in this system might uncover more species in all sites and land use types but also (1) significantly lower species richness in residential compared to forest and agricultural land uses, based on the comparatively shallow slope of the residential species accumulation curve; (2) a stronger signal of species turnover along environmental gradients, based on the large uncertainty in the GDM results; and (3) rare occurrences of *Ae. albopictus* in forest edge habitats, based on the association of this species with the urban-forest interface in Brazil (Pereira dos Santos et al. 2018; Hendy et al. 2023).

To improve understanding of how local tree cover shapes mosquito biodiversity and public health risk, future studies should aim to capture species richness and abundance along tree cover gradients and across seasons (Calderón-Arguedas et al. 2008; Troyo et al. 2008; Romero-Vega et al. 2023); assess effects of other factors in habitats that have similar tree cover but differ in aspects such as crop type or tree cover geometry; and use reforestation efforts or experimental tree cover additions at relevant spatial scales to test mosquito community responses. In the study region, tree cover responses of *Ae. aegypti* and *An. albimanus* require clarification, as we observed these regionally important vectors too rarely for statistical analysis; in particular, the canonically widespread and urban vector *Ae. aegypti* was unexpectedly rare and generalist, occurring in one site of each of the three land use types (Troyo et al. 2006; Cáceres et al. 2012). Additionally, the extent to which local tree cover effects on vector presence shape the potential for disease transmission should be tested with human pathogen surveillance in field-captured mosquitoes from different environments, and by comparison of disease case data to mosquito community data. In addition to dengue, Zika, and malaria, St. Louis Encephalitis Virus should be considered for surveillance in rural areas where humans and animals live in close proximity, because two potential vectors—*Cx. quinquefasciatus* and *Cx. nigripalpus—*were common, and this unmonitored disease is already widespread among both domesticated and wild animal hosts in Costa Rica (Medlin et al. 2016; Chaves et al. 2021a, b; Piche-Ovares et al. 2023).

## Conclusions

Overall, our findings follow patterns observed repeatedly across the globe associating tree cover with higher mosquito species richness and lower disease vector presence. We showed that tree cover both increases mosquito species richness and decreases *Ae. albopictus* presence at small spatial scales of 90–250 m. We also found that at larger spatial scales of 1000 m, warm mean annual temperatures increase habitat suitability for *Ae. albopictus* and limit tree cover contributions to species richness, but note that other factors that covary with climate across the study region may contribute to this result. Although the specific mechanisms and characteristics by which tree cover inhibits disease vectors remain unclear, the alignment of local tree cover effects on mosquito communities with other benefits for biodiversity and ecosystem services adds support to the idea that countryside landscapes can be managed to foster both human and ecosystem health.

## Supplementary Information

Below is the link to the electronic supplementary material.

Supplementary file1 (PDF 344 KB)

## Data Availability

The data presented in this manuscript are available in the DRYAD repository at 10.5061/dryad.p8cz8w9xg.

## Declarations

### Permits

Field collections and sample exports were carried out under general insect collecting permit R-036-2017-OT-CONAGEBIO, Costa Rica export permit 434-DGVS-2016, USDA veterinary import permit 134109, and Health and Human Services permit 2017-08-079.
