# Supplementary material for: Local tree cover predicts mosquito species richness and disease vector presence in a tropical countryside landscape
Source: Landsc Ecol. 2025 May 28;40(6):111. doi: 10.1007/s10980-025-02105-0 (PMC12119713; doi:10.1007/s10980-025-02105-0)
Supplement: Supplementary file 1 — Supplementary file1 (PDF 344 KB) [file 10980_2025_2105_MOESM1_ESM.pdf]

Supplemental figures and tables for "Local tree cover predicts mosquito species richness and disease vector presence in a tropical countryside landscape"
August 31, 2024

Figure S1. Species accumulation curves for each land use type were not asymptotic. Gray shading shows 95% confidence intervals.

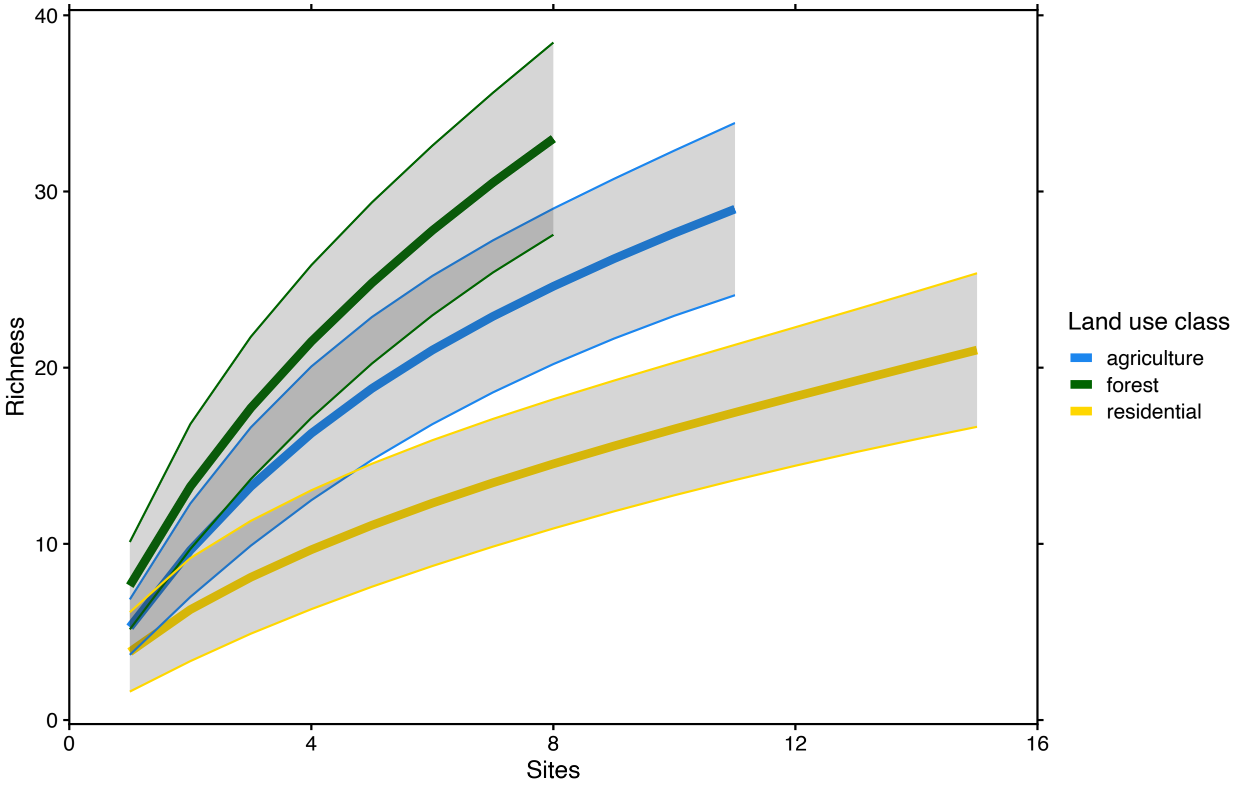

Figure S2. At the 1000 m spatial scale, species richness increased with percent tree cover only where mean annual temperatures were low to intermediate. In the warmest climates surveyed, species richness was low even at high tree cover. Points representing each survey site are colored by mean annual temperature. The blue regression line shows the results of a negative binomial GLM modeling the effect of tree cover on species richness in cooler climates (mean annual temperature ≤ 24 ºC; estimate = 2.25×10^{-2}, SE = 6.92×10^{-3}, Z = 3.26, p = 1.13×10^{-3}), and the red line shows the results for warmer climates (mean annual temperature > 24 ºC; -estimate = -3.7×10^{-2}, SE = 1.46×10^{-2}, Z = -2.53, p = 1.13×10^{-2}).

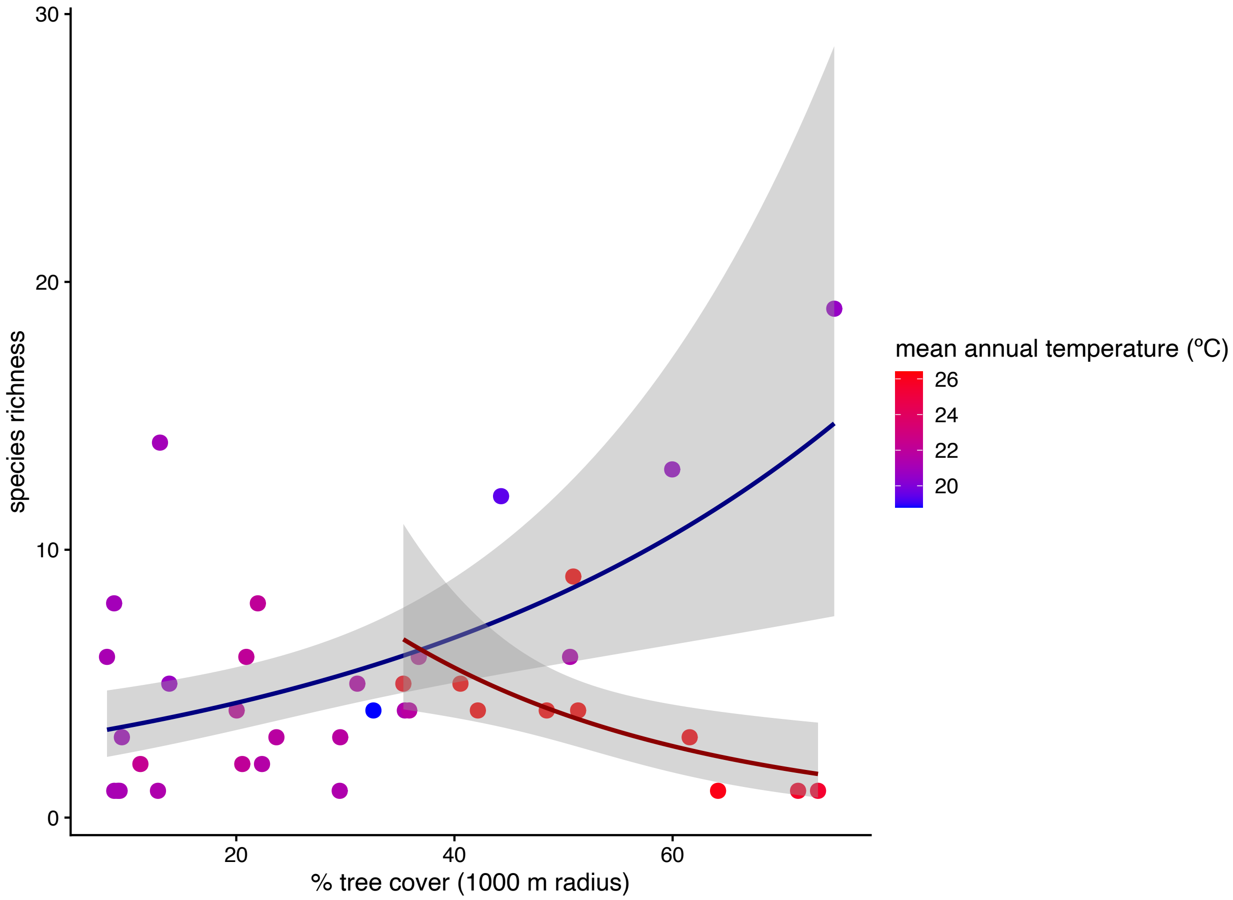

Figure S3. At the 1000 m spatial scale, *Ae. albopictus* occurrence is negatively correlated with tree cover, and positively correlated with mean annual temperature.

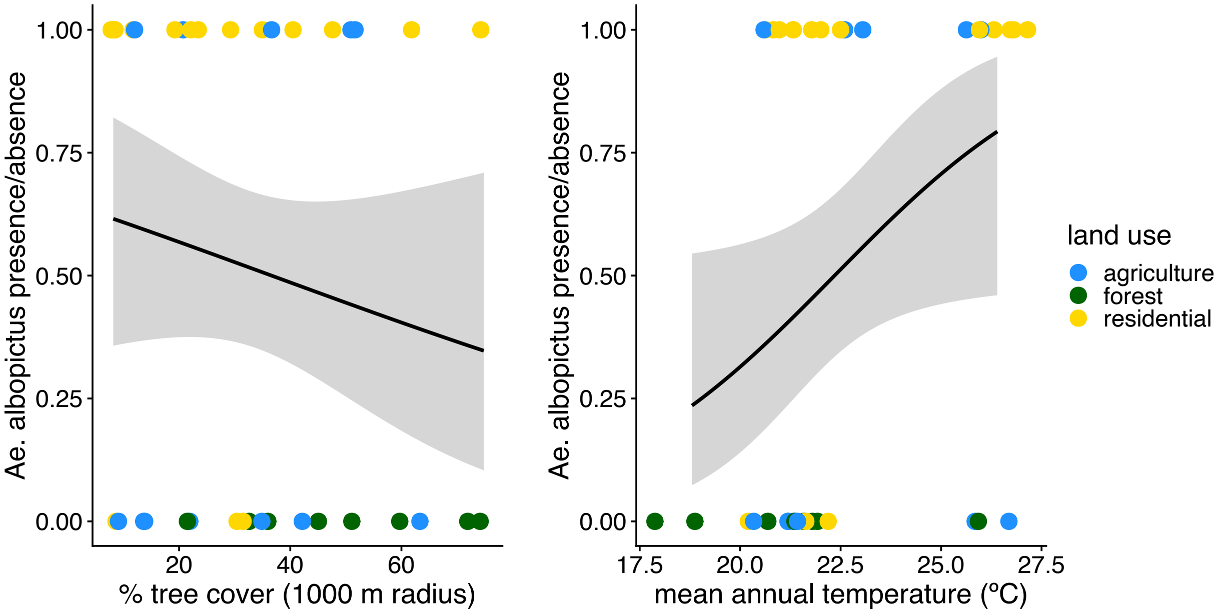

Figure S4. NMDS ordination plot of community similarity with points labeled by land use subtype. Red boxes indicate the four sites that were sampled only once, all of which were located in the geographically distinct Pavones district.

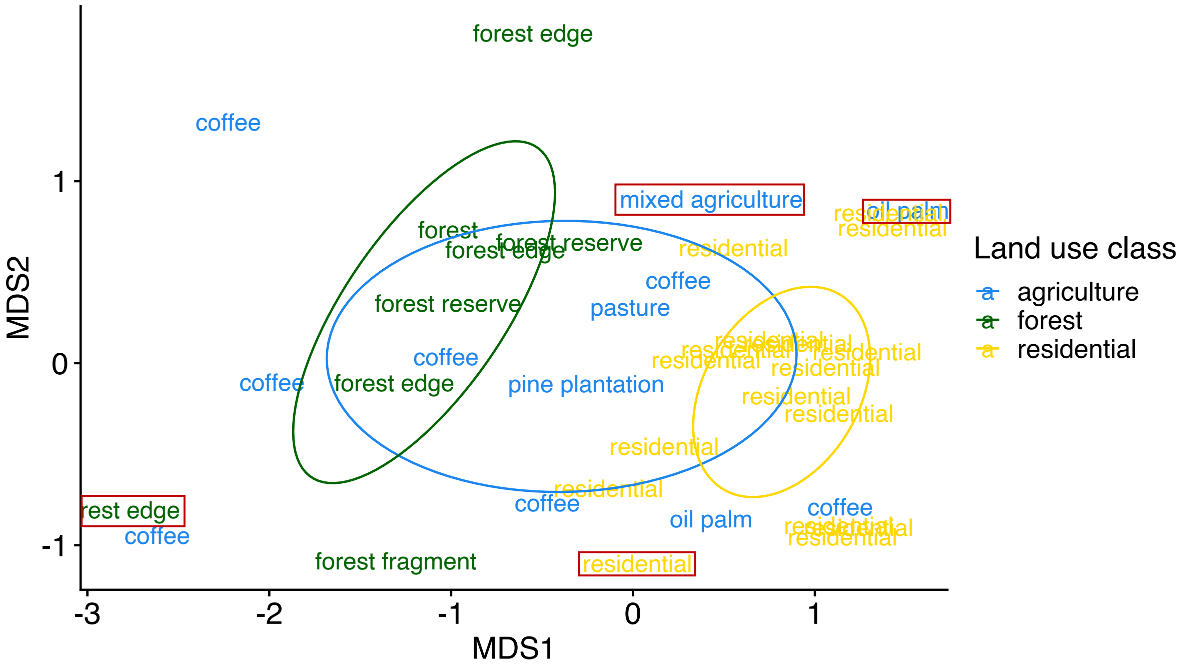

Table S1. Field survey site collection details, grouped by canton, district, and land use type.

Canton	District	Land use	Site	Collection dates (2017)	Total mosquitoes sampled	Morphologically identified *Aedes albopictus*
**Corredores**	*Ciudad Neily*	agriculture	OP03B	6/30, 7/20	32	16
			OP03	6/30, 7/20	29	12
		residential	CN04	6/30, 7/20	210	1
			CN06	6/30, 7/20	12	5
			OP01	6/30, 7/20	11	1
			CN03	6/30, 7/20	5	3
					Total = 299	*Total = 38*
**Coto Brus**	*Copabuena*	agriculture	SAFR	7/3, 7/11	7	0
			ELPU	6/26, 7/11	5	0
		residential	CB03	6/28, 7/10	37	16
			CB04	7/28, 8/3	20	2
			CB05	7/10, 8/3	5	2
			CB01	6/28, 7/10	4	2
			CB02	6/28, 7/10	4	0
					Total = 82	*Total = 22*
	Sabalito	agriculture	LOAN	6/26, 8/1	204	0
			VALE	6/27, 7/24	7	0
			FELO	6/27, 7/24	4	2
			SATE	6/22, 7/13	4	2
		forest	QUST	6/22, 7/13	3	0
		residential	SL02	7/22, 7/7	26	14
			SL01	7/22, 7/7	20	4
					Total = 268	*Total = 22*
	San Vito	agriculture	PINO	6/21, 7/13	14	6
			ISLA	6/21, 7/13	2	0
		forest	MELI	6/25, 7/31	244	0

			RIJA	6/25, 7/31	172	0
			GAPA	6/23, 7/18	97	0
			FRAG5	6/20, 7/18	20	0
			FILA	6/20, 7/17	16	0
			GABO	6/23, 7/18	12	0
		residential	SV04	7/3, 7/25	12	6
			SV06	6/20, 7/25	12	0
			SV02	6/21, 7/3	8	1
			SV05	6/21, 7/3	7	1
			SV01	6/23, 7/3	3	0
					Total = 619	*Total = 14*
**Golfito**	*Pavones*	agriculture	PV02	7/5	2	0
			PV04	7/5	2	0
		forest	PV05	7/5	1	0
		residential	PV01	7/5	11	3
					Total = 16	*Total = 3*

Table S2. Details of mosquito taxa present at each survey site, grouped by canton and land use type.

Canton	Land use	Site	Trap nights	OTU	Species
Coto Brus	residential	CB01	2	AA	*Aedes albopictus*
		CB02	2	OTU23	*Culex quinquefasciatus*
		CB03	2	OTU04	*Aedes albopictus*
				AA	*Aedes albopictus*
		CB04	2	OTU03	*Culex* sp. 1
				OTU04	*Aedes albopictus*
				OTU07	*Limatus durhamii*
				OTU09	*Culex nigripalpus*
				OTU11	*Aedes albopictus*
				OTU22	*Culex lactator*
				OTU23	*Culex quinquefasciatus*
				AA	*Aedes albopictus*
		CB05	2	OTU04	*Aedes albopictus*
			2	OTU05	*Wyeomyia adelpha/guatemala*
			2	OTU07	*Limatus durhamii*
			2	OTU08	*Orthopodomyia sp.*
			2	OTU18	*Orthopodomyia sp.*
			2	OTU19	*Orthopodomyia sp.*
			2	OTU20	*Culex coronator*
			2	OTU22	*Culex lactator*
			2	OTU30	*Limatus asulleptus*
			2	OTU51	*Orthopodomyia sp.*
			2	OTU55	*Orthopodomyia MBI-02*
			2	AA	*Aedes albopictus*
		SL01	2	OTU04	*Aedes albopictus*
				OTU11	*Aedes albopictus*
				OTU23	*Culex quinquefasciatus*
				OTU26	*Culex interrogator*
				OTU34	*Culex mollis*
				AA	*Aedes albopictus*
		SL02	2	OTU23	*Culex quinquefasciatus*
				AA	*Aedes albopictus*
		SV01	2	OTU23	*Culex quinquefasciatus*
		SV02	2	OTU04	*Aedes albopictus*
				OTU22	*Culex lactator*
				OTU23	*Culex quinquefasciatus*

			OTU57	*Culex quinquefasciatus*
			AA	*Aedes albopictus*
	SV04	2	OTU05	*Wyeomyia adelpha/guatemala*
			OTU07	*Limatus durhamii*
			OTU23	*Culex quinquefasciatus*
			OTU30	*Limatus asulleptus*
			OTU33	*Wyeomyia aporonoma*
			OTU40	*Wyeomyia undulata*
			OTU72	*Coquillettidia nigricans*
			AA	*Aedes albopictus*
	SV05	2	OTU12	*Wyeomyia complosa*
			OTU23	*Culex quinquefasciatus*
			AA	*Aedes albopictus*
	SV06	2	OTU07	*Limatus durhamii*
			OTU09	*Culex nigripalpus*
			OTU22	*Culex lactator*
			OTU23	*Culex quinquefasciatus*
			OTU72	*Coquillettidia nigricans*
agriculture	ELPU	2	OTU03	*Culex* sp. 1
			OTU15	*Trichoprosopon digitatum*
			OTU20	*Culex coronator*
			OTU35	*Trichoprosopon pallidiventer*
			OTU65	unidentified Culicidae 4
	ISLA	2	OTU15	*Trichoprosopon digitatum*
			OTU17	*Haemagogus* sp.
			OTU27	*Haemagogus* sp.
	LOAN	2	OTU03	*Culex* sp. 1
			OTU05	*Wyeomyia adelpha/guatemala*
			OTU06	*Culex nigripalpus*
			OTU07	*Limatus durhamii*
			OTU09	*Culex nigripalpus*
			OTU12	*Wyeomyia complosa*
			OTU13	*Aedes allotecnon*
			OTU14	*Wyeomyia sp. RCH-1*
			OTU15	*Trichoprosopon digitatum*
			OTU21	*Psorophora ferox*
			OTU30	*Limatus asulleptus*
			OTU41	*Aedes* sp. 1

			OTU45	*Psorophora funiculus*
			OTU52	*Wyeomyia* sp. 2
			OTU54	*Aedes allotecnon*
			OTU64	unidentified Culicidae 3
	FELO	2	AA	*Aedes albopictus*
	PINO	2	OTU05	*Wyeomyia adelpha/guatemala*
			OTU06	*Culex nigripalpus*
			OTU12	*Wyeomyia complosa*
			OTU30	*Limatus asulleptus*
			AA	*Aedes albopictus*
			AG	*Aedes aegypti*
	SAFR	2	OTU05	*Wyeomyia adelpha/guatemala*
			OTU06	*Culex nigripalpus*
			OTU23	*Culex quinquefasciatus*
	SATE	2	OTU01	*Aedes angustivittatus*
			OTU04	*Aedes albopictus*
			OTU07	*Limatus durhamii*
			OTU14	*Wyeomyia sp. RCH-1*
			OTU15	*Trichoprosopon digitatum*
			OTU17	*Haemagogus* sp.
			OTU27	*Haemagogus* sp.
			AA	*Aedes albopictus*
	VALE	2	OTU14	*Wyeomyia sp. RCH-1*
			OTU21	*Psorophora ferox*
			OTU46	*Haemagogus janthinomys*
			OTU48	*Psorophora ferox*
			OTU62	*Aedes infirmatus*
forest	FILA	2	OTU03	*Culex* sp. 1
			OTU05	*Wyeomyia adelpha/guatemala*
			OTU06	*Culex nigripalpus*
			OTU10	*Culex restrictor*
			OTU12	*Wyeomyia complosa*
			OTU13	*Aedes allotecnon*
			OTU31	*Aedes trivittatus*
			OTU41	*Aedes* sp. 1
			OTU42	*Culex* sp. 2
			OTU59	*Aedes* sp. 1
			OTU61	unidentified Culicidae 2

		OTU65	unidentified Culicidae 4
		AG	*Aedes aegypti*
FRAG5	2	OTU05	*Wyeomyia adelpha/guatemala*
		OTU06	*Culex nigripalpus*
		OTU12	*Wyeomyia complosa*
		OTU46	*Haemagogus janthinomys*
GABO	2	OTU05	*Wyeomyia adelpha/guatemala*
		OTU06	*Culex nigripalpus*
		OTU15	*Trichoprosopon digitatum*
		OTU35	*Trichoprosopon pallidiventer*
		OTU47	*Trichoprosopon sp. 3 DJS-2020*
		OTU58	*Trichoprosopon sp.*
GAPA	2	OTU12	*Wyeomyia complosa*
		OTU13	*Aedes allotecnon*
		OTU47	*Trichoprosopon sp. 3 DJS-2020*
		OTU50	*Aedes sexlineatus*
MELI	2	OTU01	*Aedes angustivittatus*
		OTU03	*Culex* sp. 1
		OTU05	*Wyeomyia adelpha/guatemala*
		OTU06	*Culex nigripalpus*
		OTU10	*Culex restrictor*
		OTU12	*Wyeomyia complosa*
		OTU13	*Aedes allotecnon*
		OTU20	*Culex coronator*
		OTU27	*Haemagogus sp.*
		OTU28	*Culex erythrothorax*
		OTU29	*unidentified Culicidae 1*
		OTU30	*Limatus asulleptus*
		OTU34	*Culex mollis*
		OTU35	*Trichoprosopon pallidiventer*
		OTU41	*Aedes* sp. 1
		OTU43	*Aedes angustivittatus*
		OTU48	*Psorophora ferox*
		OTU51	*Orthopodomyia* sp.
		OTU66	*Wyeomyia luteoventralis*

				OTU67	*Trichoprosopon sp. 5 DJS-2020*
		QUST	2	OTU05	*Wyeomyia adelpha/guatemala*
				OTU69	*Sabethes undosus*
		RIJA	2	OTU05	*Wyeomyia adelpha/guatemala*
				OTU06	*Culex nigripalpus*
				OTU09	*Culex nigripalpus*
				OTU12	*Wyeomyia complosa*
				OTU13	*Aedes allotecnon*
				OTU21	*Psorophora ferox*
				OTU23	*Culex quinquefasciatus*
				OTU24	*Wyeomyia adelpha/guatemala*
				OTU30	*Limatus asulleptus*
				OTU35	*Trichoprosopon pallidiventer*
				OTU36	*Aedes ferox*
				OTU41	*Aedes* sp. 1
				OTU43	*Aedes angustivittatus*
				OTU47	*Trichoprosopon sp. 3 DJS-2020*
				OTU48	*Psorophora ferox*
				OTU63	*Psorophora albipes*
Corredores	residential	CN03	2	OTU23	*Culex quinquefasciatus*
				AA	*Aedes albopictus*
		CN03	2	AG	*Aedes aegypti*
		CN04	2	OTU04	*Aedes albopictus*
				OTU06	*Culex nigripalpus*
				OTU07	*Limatus durhamii*
				OTU11	*Aedes albopictus*
				OTU23	*Culex quinquefasciatus*
				OTU30	*Limatus asulleptus*
				OTU53	*Aedes albopictus*
				AA	*Aedes albopictus*
		CN06	2	AA	*Aedes albopictus*
		OP01	2	OTU06	*Culex nigripalpus*
				OTU22	*Culex lactator*
				OTU23	*Culex quinquefasciatus*
				AA	*Aedes albopictus*
	agriculture	OP03	2	OTU01	*Aedes angustivittatus*

				OTU06	*Culex nigripalpus*
				OTU21	*Psorophora ferox*
				OTU23	*Culex quinquefasciatus*
				OTU25	*Mansonia titillans*
				OTU28	*Culex erythrothorax*
				OTU39	*Anopheles albimanus*
				OTU66	*Wyeomyia luteoventralis*
				AA	*Aedes albopictus*
		OP03B	2	OTU01	*Aedes angustivittatus*
				OTU07	*Limatus durhamii*
				OTU40	*Wyeomyia undulata*
				AA	*Aedes albopictus*
Golfito	residential	PV01	1	OTU05	*Wyeomyia adelpha/guatemala*
				OTU25	*Mansonia titillans*
				OTU28	*Culex erythrothorax*
				OTU58	*Trichoprosopon sp.*
				AA	*Aedes albopictus*
	agriculture	PV02	1	OTU01	*Aedes angustivittatus*
				OTU07	*Limatus durhamii*
				OTU23	*Culex quinquefasciatus*
				OTU35	*Trichoprosopon pallidiventer*
		PV04	1	OTU23	*Culex quinquefasciatus*
	forest	PV05	1	OTU15	*Trichoprosopon digitatum*

Table S3. Number of sites in each land use category where each mosquito species was present.

Species	Agriculture	Forest	Residential	Total
Aedes albopictus	5	0	13	18
Culex quinquefasciatus	4	1	12	17
Culex nigripalpus	4	5	4	13
Wyeomyia adelpha/guatemala	3	6	3	12
Limatus durhamii	3	0	5	9
Wyeomyia complosa	2	5	1	8
Limatus asulleptus	2	2	3	7
Aedes angustivittatus	4	2	0	6
Trichoprosopon digitatum	4	2	0	6
Aedes allotecnon	1	4	0	5
Culex lactator	0	0	5	5
Culex sp. 1	2	2	1	5
Psorophora ferox	3	2	0	5
Trichoprosopon pallidiventer	2	3	0	5
Aedes sp. 1	1	3	0	4
Aedes aegypti	1	1	1	3
Culex coronator	1	1	1	3
Culex erythrothorax	1	1	1	3
Haemagogus sp.	2	1	0	3
Trichoprosopon sp. 3 DJS-2020	0	3	0	3
Wyeomyia sp. RCH-1	3	0	0	3
Coquillettidia nigricans	0	0	2	2
Culex mollis	0	1	1	2
Culex restrictor	0	2	0	2
Haemagogus janthinomys	1	1	0	2
Mansonia titillans	1	0	1	2
Orthopodomyia sp.	0	1	1	2
Trichoprosopon sp. 1	0	1	1	2
unidentified Culicidae 4	1	1	0	2
Wyeomyia luteoventralis	1	1	0	2
Wyeomyia undulata	0	0	1	2
Aedes ferox	0	1	0	1
Aedes infirmatus	1	0	0	1
Aedes sexlineatus	0	1	0	1
Aedes trivittatus	0	1	0	1

Anopheles albimanus	1	0	0	1
Culex interrogator	0	0	1	1
Culex sp. 2	0	1	0	1
Orthopodomyia MBI-02	0	0	1	1
Psorophora albipes	0	1	0	1
Psorophora funiculus	1	0	0	1
Sabethes undosus	0	1	0	1
Trichoprosopon sp. 5 DJS-2020	0	1	0	1
unidentified Culicidae 1	0	1	0	1
unidentified Culicidae 2	0	1	0	1
unidentified Culicidae 3	1	0	0	1
Wyeomyia aporonoma	0	0	1	1
Wyeomyia sp. 2	1	0	0	1

Table S4. Results of generalized linear models estimating the effects of tree cover across spatial scales on species richness. Significant models are highlighted with bold text.

Tree cover radius (m)	Estimated effect	Standard error	Z-value	p-value	AIC
30	3.67×10^{-3}	3.57×10^{-3}	1.03	3.03×10^{-1}	194.88
40	5.13×10^{-3}	3.72×10^{-3}	1.38	1.77×10^{-1}	194.10
50	5.50×10^{-3}	3.85×10^{-3}	1.43	1.54×10^{-1}	193.98
60	6.64×10^{-3}	4.02×10^{-3}	1.65	9.8×10^{-2}	193.24
70	7.21×10^{-3}	4.01×10^{-3}	1.80	7.23×10^{-2}	192.76
80	8.02×10^{-3}	4.13×10^{-3}	1.94	5.24×10^{-2}	192.18
**90**	**8.93×10^{-3}**	**4.25×10^{-3}**	**2.10**	**3.58×10^{-2}**	**191.45**
**100**	**9.13×10^{-3}**	**4.30×10^{-3}**	**2.12**	**3.39×10^{-2}**	**191.33**
**110**	**9.64×10^{-3}**	**4.37×10^{-3}**	**2.20**	**2.75×10^{-2}**	**190.96**
**120**	**1.05×10^{-2}**	**4.46×10^{-3}**	**2.36**	**1.84×10^{-2}**	**190.24**
**130**	**1.06×10^{-2}**	**4.47×10^{-3}**	**2.37**	**1.76×10^{-2}**	**190.15**
**140**	**1.10×10^{-2}**	**4.58×10^{-3}**	**2.39**	**1.66×10^{-2}**	**190.03**
**150**	**1.17×10^{-2}**	**4.62×10^{-3}**	**2.53**	**1.16×10^{-2}**	**189.40**
**160**	**1.20×10^{-2}**	**4.67×10^{-3}**	**2.58**	**9.92×10^{-3}**	**189.13**
**170**	**1.24×10^{-2}**	**4.69×10^{-3}**	**2.64**	**8.18×10^{-3}**	**188.79**
**180**	**1.29×10^{-2}**	**4.77×10^{-3}**	**2.70**	**7.01×10^{-3}**	**188.53**
**190**	**1.30×10^{-2}**	**4.83×10^{-3}**	**2.69**	**7.09×10^{-3}**	**188.49**
**200**	**1.35×10^{-2}**	**4.87×10^{-3}**	**2.77**	**5.59×10^{-3}**	**188.11**
**250**	**1.40×10^{-2}**	**5.00×10^{-3}**	**2.80**	**5.08×10^{-3}**	**187.91**
**300**	**1.37×10^{-2}**	**5.01×10^{-3}**	**2.73**	**6.27×10^{-3}**	**188.21**
**350**	**1.38×10^{-2}**	**5.09×10^{-3}**	**2.72**	**6.57×10^{-3}**	**188.27**
**400**	**1.37×10^{-2}**	**5.24×10^{-3}**	**2.62**	**8.79×10^{-3}**	**188.76**
**450**	**1.33×10^{-2}**	**5.36×10^{-3}**	**2.47**	**1.34×10^{-2}**	**189.47**
**500**	**1.26×10^{-2}**	**5.48×10^{-3}**	**2.30**	**2.17×10^{-2}**	**190.29**
**550**	**1.19×10^{-2}**	**5.54×10^{-3}**	**2.15**	**3.13×10^{-2}**	**190.94**
**600**	**1.15×10^{-2}**	**5.58×10^{-3}**	**2.06**	**3.93×10^{-2}**	**191.33**
**650**	**1.11×10^{-2}**	**5.63×10^{-3}**	**1.98**	**4.80×10^{-2}**	**191.70**
700	1.09×10^{-2}	5.68×10^{-3}	1.92	5.51×10^{-2}	191.97
750	1.06×10^{-2}	5.73×10^{-3}	1.85	6.43×10^{-2}	192.26
800	1.03×10^{-2}	5.77×10^{-3}	1.78	7.44×10^{-2}	192.56
850	1.01×10^{-2}	5.84×10^{-3}	1.73	8.38×10^{-2}	192.81
900	9.82×10^{-3}	5.90×10^{-3}	1.66	9.64×10^{-2}	193.08
950	9.49×10^{-3}	5.98×10^{-3}	1.59	1.13×10^{-1}	193.35
1000	9.23×10^{-3}	6.07×10^{-3}	1.52	1.28×10^{-1}	193.57

Table S5. Generalized linear model results estimating effects of percent tree cover calculated at a 1000m radius around survey sites and mean annual temperature on mosquito species richness.

Environmental variable	Estimate	Standard Error	Z-value	p-value
% tree cover at 1000 m radius	5.96×10^{-3}	6.24×10^{-3}	0.96	0.34
Mean annual temperature	3.18	7.10×10^{-2}	-4.0×10^{-3}	0.99
Tree cover * temperature	-9.48×10^{-3}	3.48×10^{-3}	-2.72	6.48×10^{-3} *

Table S6. Results of generalized linear models estimating the effects of tree cover across spatial scales on *Aedes albopictus* presence. Significant models are highlighted with bold text.

Tree cover radius (m)	Estimated effect	Standard error	Z-value	p-value	AIC
**30**	**-2.61×10^{-2}**	**1.19×10^{-2}**	**-2.19**	**2.88×10^{-2}**	**49.28**
**40**	**-2.95×10^{-2}**	**1.31×10^{-2}**	**-2.25**	**2.46×10^{-2}**	**48.70**
**50**	**-3.61×10^{-2}**	**1.47×10^{-2}**	**-2.46**	**1.39×10^{-2}**	**46.80**
**60**	**-3.36×10^{-2}**	**1.44×10^{-2}**	**-2.33**	**1.98×10^{-2}**	**48.21**
**70**	**-3.51×10^{-2}**	**1.48×10^{-2}**	**-2.37**	**1.76×10^{-2}**	**47.79**
**80**	**-4.00×10^{-2}**	**1.63×10^{-2}**	**-2.45**	**1.44×10^{-2}**	**46.84**
**90**	**-4.15×10^{-2}**	**1.69×10^{-2}**	**-2.46**	**1.40×10^{-2}**	**46.85**
**100**	**-4.09×10^{-2}**	**1.72×10^{-2}**	**-2.38**	**1.72×10^{-2}**	**47.32**
**110**	**-4.46×10^{-2}**	**1.83×10^{-2}**	**-2.44**	**1.47×10^{-2}**	**46.60**
**120**	**-4.56×10^{-2}**	**1.88×10^{-2}**	**-2.43**	**1.52×10^{-2}**	**46.74**
**130**	**-4.52×10^{-2}**	**1.88×10^{-2}**	**-2.41**	**1.61×10^{-2}**	**46.91**
**140**	**-4.68×10^{-2}**	**1.96×10^{-2}**	**-2.39**	**1.69×10^{-2}**	**46.90**
**150**	**-4.63×10^{-2}**	**1.95×10^{-2}**	**-2.37**	**1.79×10^{-2}**	**47.25**
**160**	**-4.71×10^{-2}**	**2.00×10^{-2}**	**-2.36**	**1.83×10^{-2}**	**47.28**
**170**	**-4.73×10^{-2}**	**2.03×10^{-2}**	**-2.33**	**1.96×10^{-2}**	**47.43**
**180**	**-4.57×10^{-2}**	**2.02×10^{-2}**	**-2.27**	**2.34×10^{-2}**	**48.09**
**190**	**-4.52×10^{-2}**	**2.03×10^{-2}**	**-2.23**	**2.60×10^{-2}**	**48.40**
**200**	**-4.62×10^{-2}**	**2.08×10^{-2}**	**-2.22**	**2.63×10^{-2}**	**48.40**
**250**	**-4.36×10^{-2}**	**2.10×10^{-2}**	**-2.08**	**3.75×10^{-2}**	**49.41**
300	-3.90×10^{-2}	1.99×10^{-2}	-1.96	5.00×10^{-2}	50.30
350	-3.67×10^{-2}	1.95×10^{-2}	-1.89	5.94×10^{-2}	50.82
400	-3.52×10^{-2}	1.92×10^{-2}	-1.83	6.71×10^{-2}	51.18
450	-3.40×10^{-2}	1.89×10^{-2}	-1.80	7.24×10^{-2}	51.41
500	-3.35×10^{-2}	1.87×10^{-2}	-1.79	7.29×10^{-2}	51.48
550	-3.25×10^{-2}	1.84×10^{-2}	-1.77	7.69×10^{-2}	51.64
600	-3.01×10^{-2}	1.79×10^{-2}	-1.68	9.25×10^{-2}	52.06
650	-2.77×10^{-2}	1.75×10^{-2}	-1.58	1.13×10^{-1}	52.49
700	-2.52×10^{-2}	1.72×10^{-2}	-1.47	1.43×10^{-1}	52.94
750	-2.26×10^{-2}	1.69×10^{-2}	-1.34	1.81×10^{-1}	53.36
800	-2.10×10^{-2}	1.68×10^{-2}	-1.25	2.12×10^{-1}	53.62
850	-1.96×10^{-2}	1.67×10^{-2}	-1.17	2.41×10^{-1}	53.83
900	-1.85×10^{-2}	1.67×10^{-2}	-1.10	2.70×10^{-1}	54.00
950	-1.75×10^{-2}	1.68×10^{-2}	-1.04	2.98×10^{-1}	54.15
1000	-1.65×10^{-2}	1.69×10^{-2}	-0.98	3.28×10^{-1}	54.28

Table S7. Generalized linear model results estimating effects of percent tree cover calculated at a 1000m radius around survey sites and mean annual temperature on *Ae. albopictus* presence.

Environmental variable	Estimate	Standard Error	Z-value	p-value
% tree cover at 1000 m radius	-8.34×10^{-2}	3.61×10^{-2}	-2.31	0.021 *
Mean annual temperature	9.37×10^{-1}	4.20×10^{-2}	2.23	0.026 *
Tree cover * temperature	-3.46×10^{-3}	1.54×10^{-2}	-0.225	0.822

Table S8. Generalized dissimilarity modeling results for mosquito community turnover along environmental gradients of mean annual temperature, elevation, geographic distance, and tree cover calculated for different radii surrounding survey sites.

	sum of coefficients					
**Tree cover radius (m)**	**percent tree cover**	**mean annual temperature**	**geographic distance**	**elevation**	**intercept**	**percent deviance explained**
30	0.659	0.916	0.410	0	1.64	5.430
40	0.602	0.890	0.417	0	1.64	5.727
50	0.725	0.858	0.394	0	1.60	6.797
60	0.698	0.904	0.420	0	1.63	6.046
70	0.694	0.900	0.408	0	1.63	6.036
80	0.750	0.875	0.388	0	1.61	6.472
90	0.737	0.880	0.409	0	1.61	6.367
100	0.710	0.889	0.399	0	1.62	6.110
110	0.788	0.873	0.372	0	1.59	6.651
120	0.801	0.878	0.376	0	1.59	6.643
130	0.823	0.875	0.356	0	1.59	6.803
140	0.840	0.873	0.349	0	1.59	6.745
150	0.831	0.872	0.347	0	1.59	6.590
160	0.850	0.868	0.333	0	1.59	6.566
170	0.837	0.865	0.322	0	1.60	6.393
180	0.799	0.860	0.340	0	1.62	5.821
190	0.773	0.858	0.357	0	1.65	5.472
200	0.792	0.850	0.356	0	1.65	5.422
250	0.628	0.856	0.455	0	1.74	4.383
300	0.520	0.880	0.520	0	1.79	4.106
350	0.499	0.884	0.527	0	1.79	4.094
400	0.465	0.889	0.513	0	1.79	4.095
450	0.434	0.905	0.515	0	1.79	3.947
500	0.405	0.921	0.517	0	1.79	3.812
550	0.372	0.937	0.525	0	1.80	3.690
600	0.314	0.543	0.950	0	1.80	3.544
650	0.258	0.558	0.962	0	1.81	3.424
700	0.211	0.571	0.973	0	1.81	3.339
750	0.163	0.583	0.982	0	1.82	3.272
800	0.119	0.593	0.990	0	1.82	3.224
850	0.066	0.608	0.999	0	1.83	3.184
900	0.023	0.623	1.004	0	1.83	3.166
950	0.001	0.631	1.006	0	1.84	3.163
1000	0	0.631	1.007	0	1.84	3.163
